# Microbial Communities and Environmental Factors Interact to Regulate Soil Respiration Under Nitrogen Addition Conditions in Alpine Meadows in Northwest China

**DOI:** 10.3390/microorganisms13092098

**Published:** 2025-09-09

**Authors:** Xiaojuan Cao, Jinlong Wang, Bota Bahethan, Yudong Chen, Junjie Liu, Guanghui Lü

**Affiliations:** 1 College of Ecology and Environment, Xinjiang University, Urumqi 830017, China; cxj019085@163.com (X.C.); bota119@xju.edu.cn (B.B.); cyd666@stu.xju.edu.cn (Y.C.); liujunjie@xju.edu.cn (J.L.); ler@xju.edu.cn (G.L.); 2 Key Laboratory of Oasis Ecology of Education Ministry, Xinjiang University, Urumqi 830017, China; 3 Xinjiang Jinghe Observation and Research Station of Temperate Desert Ecosystem, Ministry of Education, Jinghe 833300, China

**Keywords:** alpine meadow, nitrogen addition, soil respiration, soil enzyme activity, soil microbial community structure

## Abstract

Alpine meadow ecosystems are highly sensitive to global change, yet the response mechanisms of soil respiration (Rs) to nitrogen deposition remain unclear. This research employed a gradient nitrogen addition experiment (0, 5, 10, 15, 20 g·m^−2^·a^−1^) in an alpine meadow ecosystem in Northwest China to determine the major factors regulating soil respiration responses. High nitrogen inputs (N15 and N20) significantly elevated Rs by 31.96% and 29.21% relative to the control (*p* < 0.05). Nitrogen addition significantly increased soil ammonium nitrogen (NH_4_^+^-N) content, as well as the activities of cellobiohydrolase (CBH) and peroxidase (POD). Microbial community structure shifted with nitrogen addition, showing increased relative abundance of Actinobacteriota (14–25%) and Basidiomycota (13–26%). Functional prediction analysis revealed that high nitrogen treatments enhanced bacterial carbon metabolism functions such as fermentation and ureolysis, while enriching fungal functional guilds like Wood Saprotroph and Arbuscular Mycorrhizal fungi. Partial Least Squares Path Modeling (PLS-PM) indicated that nitrogen addition indirectly drives changes in Rs by regulating physicochemical factors (e.g., NH_4_^+^-N), which subsequently influence microbial community composition, functional potential, and key enzyme activities. These findings elucidate the factors influencing soil respiration under varying nitrogen addition levels, providing a theoretical basis for assessing soil carbon cycling in alpine meadows under global change scenarios.

## 1. Introduction

Fossil fuel combustion and agricultural and industrial activities have markedly increased global nitrogen deposition since the Industrial Revolution [1,2]. The nutrient imbalances from external nitrogen inputs significantly impact the global carbon cycle in terrestrial ecosystems [3]. Nitrogen, as a limiting element for plant growth and soil microbial metabolism, can promote plant development and enhance soil carbon storage. Its exogenous input stimulates plant growth and soil organic carbon sequestration, but may also alter substrate availability, microbial community composition, and enzyme activity, thereby inducing a “priming effect” that promotes soil respiration and accelerates the decomposition of soil organic carbon pools, ultimately reducing soil fertility [3,4]. Therefore, the effects of nitrogen deposition on soil respiration depend not only on the dynamics of soil carbon pools but also on the coupled regulation of environmental factors and microorganisms.

Soil respiration is crucial for regulating the terrestrial carbon cycle and soil carbon reservoirs, representing a significant carbon flux in terrestrial ecosystems, second only to photosynthesis [5,6]. Moreover, it is a primary source of atmospheric carbon and is key to the carbon cycle in grassland ecosystems [7]. Nitrogen deposition impacts soil respiration by changing the availability of soil nutrients and the supply of carbon. Extensive research in China and worldwide has examined the impact of nitrogen addition on soil respiration [5], the findings remain inconsistent. Some studies reported that nitrogen addition stimulates soil respiration due to increased substrate supply or enhanced root respiration [6]. In contrast, other studies observed that under high nitrogen inputs, soil microbes may shift from nitrogen limitation to carbon limitation, reducing microbial activity, suppressing the production of lignin-degrading enzymes, and causing soil acidification, thereby inhibiting respiration [8,9]. Still, other research found no significant effect [10]. Moreover, increased nitrogen inputs stimulate root respiration, which offsets the reduction in microbial respiration caused by nitrogen addition, thereby mitigating the overall impact of elevated soil nitrogen on soil respiration [11]. The inconsistency in results may be attributed to differences in ecosystem type, environmental conditions, nitrogen addition level, fertilizer type, and experimental duration [12,13]. A major source of uncertainty lies in the insufficient integration of microbial ecological mechanisms into interpretations of Rs responses.

The diverse array of soil microorganisms forms a complex food web, acts as primary decomposers in terrestrial ecosystems, and represents a direct source of soil enzyme activities essential for key geochemical cycles, such as soil respiration [14]. Nutrient inputs can substantially alter the structure and function of soil microbial communities, and nitrogen addition has been shown to induce shifts in bacterial and fungal composition [15,16,17]. For instance, fungi are generally more sensitive than bacteria to changes in nutrient inputs and availability [18]. Nitrogen addition influences soil organic carbon decomposition by modifying the abundance of functional genes involved in the carbon cycle and the associated enzyme activities, thereby affecting CO_2_ emissions [19]. Differences in the effects of nitrogen on soil respiration may arise from compositional changes in the microbial community induced by nitrogen additions [20], as well as from variations in carbon fixation and allocation across ecosystems [6]. Soil extracellular enzymes, including hydrolytic and oxidative types, catalyze the decomposition and mineralization of SOC, thereby facilitating nutrient cycling, and their activities are shaped by ecosystem location, initial N status, and other factors, which increases the uncertainty about the response mechanism of soil respiration under N addition [21,22]. Short-term N additions can cause rapid but sometimes transient changes: low-level N inputs often stimulate Rs by alleviating N limitation and increasing microbial biomass, whereas high-level inputs may suppress Rs through carbon limitation, reduced diversity, and decreased lignin-degrading enzyme abundance [19,23]. Although numerous studies have examined the effects of N addition on Rs, the direct and indirect pathways—mediated by changes in soil physicochemical properties, microbial community composition, and enzyme activities—remain incompletely understood.

Alpine meadows are highly sensitive to human activities and global change, with nitrogen deposition increasingly affecting their soil carbon processes [23]. Nitrogen deposition modifies the dynamics of soil organic matter by affecting soil physicochemical properties and microbial community life history strategies, leading to increased carbon emissions and accelerating the recovery of degraded alpine meadows [18]. The Bayinbruk Grassland in Xinjiang represents a prototypical temperate alpine meadow ecosystem. Since 2020, we have established a long-term N addition experiment at this site, applying NH_4_NO_3_ at different levels annually (2020–2023) to simulate sustained atmospheric N deposition. This long-term experiment provides an opportunity to investigate the mechanisms driving Rs responses under varying N inputs, integrating microbial community composition, enzyme activity, and soil physicochemical properties.

Understanding how nitrogen addition influences soil carbon pools is crucial for managing alpine meadows and similar ecosystems. In this study, we used the Bayinbruk alpine meadow as the research object and performed 16S rDNA high-throughput sequencing to reveal the factors affecting soil respiration at different nitrogen addition levels. The goal was to provide parameters for future research, enhance modeling and prediction capabilities for alpine meadow ecosystems’ response to atmospheric nitrogen deposition, and establish a theoretical foundation for developing empirically grounded management strategies for grassland ecosystems. The following research issues were investigated in this study: (1) How do varying degrees of nitrogen input affect soil respiration in alpine meadows? (2) What biotic and abiotic factors influence the respiration of soil? and (3) How do the microbial populations, soil enzyme activity, and physicochemical properties of the soil interact to influence soil respiration? The findings will bolster research on the carbon balance and the long-term viability of alpine grassland ecosystems.

## 2. Materials and Methods

### 2.1. Study Site

**Figure 1:**
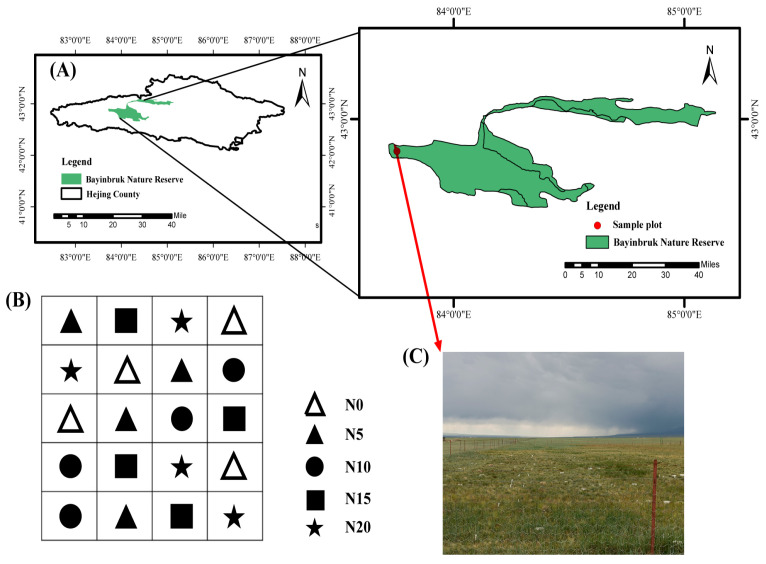
Schematic diagram of the location of the study area and sampling sites. (**A**) Location of Bayinbruk Nature Reserve; (**B**) Coding system of sampling plots (N0–N20); (**C**) Field photograph showing a representative sampling plot within the reserve.

The Bayinbruk Grassland Ecosystem Research Station (42°53′ N, 83°42′ E, elevation 2470 m a.s.l.), situated in Hejing County, Bayin’guoleng Mongol Autonomous Prefecture, Xinjiang, China, is located close to the research region [24] (Figure 1). This region experiences an average temperature of −4.8 °C, with January having the lowest temperature of −48 °C. The annual evaporation ranges from 1022.9 to 1247.5 mm, whereas the mean precipitation is 276.2 mm. The region has a typical high alpine climate with little precipitation, considerable evaporation, and no absolute frost-free season. The soil type is categorized as Calcic Kastanozems (World Reference Base for Soil Resources, WRB). Carex rhynchophysa is the predominant vegetative species in the study region. *Festuca ovina* L., *Agropyron cristatum* (L.) Gaertn, *Poa pratensis* L., and *Potentilla bifurca* Linn are the principal established species [25].

### 2.2. Experimental Design

Five nitrogen addition treatments were established in 2020 according to the nitrogen deposition levels recorded for Bayinbruk Grassland in previous years [25,26]. This experiment used a randomized block group experimental design and in situ simulations. These treatments were as follows: 0, 5, 10, 15, and 20 g·m^−2^·a^−1^ (defined as N0, N5, N10, N15 and N20, respectively). Each treatment had four replicates, resulting in a total of 20 plots, each measuring 3 m × 3 m. A buffer strip (0.5 m) was set up to exclude disturbances between the neighboring sample plots. Ammonium nitrate (NH_4_NO_3_, 35% nitrogen content) was used as the sole nitrogen source and was evenly broadcast by hand at the end of April each year from 2020 to 2023.

To avoid transient effects immediately after fertilization and focus on the integrated influence of N addition on microbial activity under steady-state conditions, Rs measurements were conducted after a sufficient interval following N application, in line with previous studies [27]. Soil samples were collected in late August 2023, corresponding to the main growing season of the alpine meadow. At this time, soil respiration and nitrogen cycling are most active [28]; however, this study does not cover seasonal variations throughout the year, mainly reflecting the dynamics of the growing season.

During sampling, one 0.5 m × 0.5 m subplot was chosen randomly from each sample plot, all vegetation within the tiny sample plot was harvested flush to the ground, and the surface fall was removed. Three soil samples were obtained from each sample plot, and the top 0–10 cm of the soil was collected using the soil auger method (diameter: 5 cm). These samples were evenly mixed and run through a 2 mm sieve to eliminate tiny roots and stones. A portion of the mixed soil was air-dried at ambient temperature to analyze its physicochemical properties, while another portion was preserved at 4 °C to measure the activity of soil enzymes and soil microorganisms [29].

### 2.3. Determination of Soil Physicochemical Properties

The pH was determined by potentiometric method on a 1:5 soil:water ratio suspension (PHS-3C, Shanghai Yidian Scientific Instrument Co., Ltd., Shanghai, China) [30]. Electrical conductivity (EC) was determined by conductivity method (DDS-307, Shanghai Yidian Scientific Instrument Co., Ltd., Shanghai, China) [30]. The potassium dichromate-heating technique [31] was used to assess soil organic carbon (SOC); Soil total nitrogen (TN) content was determined by the Kjeldahl digestion method [32]; soil total P (TP) content was determined by the H_2_SO_4_-HClO_4_ method using spectrophotometry [33]. The molybdenum antimony colorimetric technique and NaHCO_3_ leaching were used to estimate the available phosphorus (AP) in the soil. Soil ammonium nitrogen (NH_4_^+^-N) was evaluated using the indophenol blue colorimetric method, while soil nitrate nitrogen (NO_3_^−^-N) was assessed using the ultraviolet spectrophotometric method [34].

### 2.4. Measurement of Soil Enzyme Activities

Using four replicates, a 96-microtiter enzyme label plate fluorescence analysis was used to assess the activities of soil enzymes [35]. To create the soil suspension, 1.00 ± 0.02 g of soil samples were put in a 250 mL shaking container, 100 mL of 50 mmol L-1 sodium acetate buffer was added, and a tiny glass bead was mixed. The mixture was then shaken for 1 h (25 °C 180 rpm). The suspension was then placed on a magnetic stirrer at 600–1000 rpm with constant stirring. For the different experiments, 200 μL of soil suspension in the 96-well microplates was mixed with seven hydrolase substrates (50 μL) there are involved in soil C, N, and P cycles (Table S1) were added to as the sample control; 50 μL of enzyme-substrate and 200 μL of soil suspension were used as the sample control; 50 μL of sodium acetate buffer and 200 μL of soil suspension were used as the control; and 50 μL of the standard substrate and 200 μL of soil suspension was used as the quench control. After 4 h of dark incubation at 25 °C, the reaction was stopped by adding 10 μL of 0.5 N NaOH. The fluorescence values were determined using a multifunctional enzyme marker (Tecan, Männedorf, Switzerland), excited at 365 nm for hydrolases and 450 nm for oxidases [36]. The rate at which 1 g of dry soil converted to substrate in 1 h (μmol h^−1^ g^−1^) represented the samples’ level of enzyme activity.

### 2.5. Soil Respiration Measurements

A PVC ring (15 cm height and 20 cm inner diameter) was placed in each plot approximately 10 cm into the soil and 5 cm above the ground surface to evaluate soil respiration. This was performed in May 2023. The day before measurement, weeds inside the ring were cut using scissors to sever any carbon flow from aboveground plants to the root system. During the growing season in 2023 (August), soil respiration was measured on clear days using an LI-8100 automated soil CO_2_ flux system (LI-COR, Lincoln, NE, USA). All experimental measurements occurred between 10:00 and 12:00 local time on the same day, aiming to accurately reflect average daily CO_2_ emissions [37]. Throughout each measurement session, soil temperature (ST) was recorded at a 10 cm depth adjacent to the PVC collar, utilizing a temperature sensor (MS-10, Rain Root Technology Co., Ltd., Beijing, China).

Rs fluxes were calculated from CO_2_ accumulation in the chamber using the LI-8100 software (v4.0.0) according to the manufacturer’s instructions. The LI-8100 system employs a closed non-steady state transient method to measure soil CO_2_ flux, estimating the diffusion rate of CO_2_ into free air outside the chamber by analyzing the rate of increase inb CO_2_ concentration within the chamber [38]. This method allows for temperature corrections and provides accurate Rs measurements under varying environmental conditions. Data quality was further ensured by excluding measurements with abnormal CO_2_ increase patterns or signal fluctuations, following standard protocols [27,37].

### 2.6. Soil Microbiological Sequencing

Microbiological analyses were conducted at Beijing Novozymes Technology Co. (Beijing, China) DNA was extracted from soil samples using the NEBNext^®^ DNA Library Prep Kit (New England Biolabs, Inc., Ipswich, MA, USA) following the manufacturer’s instructions, with 0.1 μg of DNA obtained from each sample. Bacterial diversity was assessed using primers targeting the 16SV4 region (515F and 806R); eukaryotic microorganisms were analyzed using 18SV4 region primers (528F and 706R); and fungal diversity was determined with ITS1 region primers (ITS5–1737F and ITS2–2043R). The amplified regions for bacteria included 16SV3–V4, 16SV4–V5, and 16SV5–V7, while archaea were amplified with 16SV4–V5, 16SV8, and 18SV9, and fungi with ITS2 [39]. Sequencing involved paired-end readings with distinct barcodes trimmed to remove primer and barcode sequences. Based on a sequence number standard from the sample with the fewest sequences, the absolute abundance of ASVs was normalized. On the normalized data, alpha and beta diversity analyses were carried out.

### 2.7. Statistical Analyses

To examine the differences between treatments at various amounts of nitrogen addition, one-way analysis of variance (ANOVA) and least significant difference (LSD) analyses were conducted, with significant differences identified at *p* < 0.05. The results are displayed as the “mean ± standard error.” A histogram showing the relative abundance distribution of the top 10 species for each sample at various taxonomic levels (phylum, order, family, genus, and species) was generated using the SVG function in Perl. Using QIIME v.2.0 software, microbial diversity indicators such as the Shannon and Simpson indices were computed. The ranking of soil microorganisms and soil factors was conducted using the vegan package in R (version 4.3.0), with redundancy analysis (RDA) used to further explore correlations between environmental factors and species abundance.

Meanwhile, soil respiration (Rs), soil physicochemical properties (ST, pH, EC, TN, TP, AP, SOC, NO_3_^−^-N, NH_4_^+^-N), soil enzyme activities (α-G, β-G, CBH, β-1, NAG, LAP, ALP, POD), and soil microbial diversity indices (Shannon index, Simpson index) were selected and analyzed in R (4.3.1) using the factoextra software package for multiple factor analysis (MFA) to explore the interrelationships between multiple sets of variables. Key parameters were selected to represent specific variables: SOC, NO_3_^−^-N, and NH_4_^+^-N for soil physicochemical properties; CBH, NAG, and POD for soil enzyme activities; and the Shannon and Simpson indices for bacterial and fungal diversity. A partial least squares path model (PLS-PM) was constructed using the plspm package in R (version 4.3.1) to further examine the interactions between nitrogen addition levels, soil characteristics, enzyme activities, and microbial communities on soil respiration.

## 3. Results

### 3.1. Soil Respiration and Soil Abiotic Factors Are Affected by Different Levels of Nitrogen Addition

**Figure 2:**
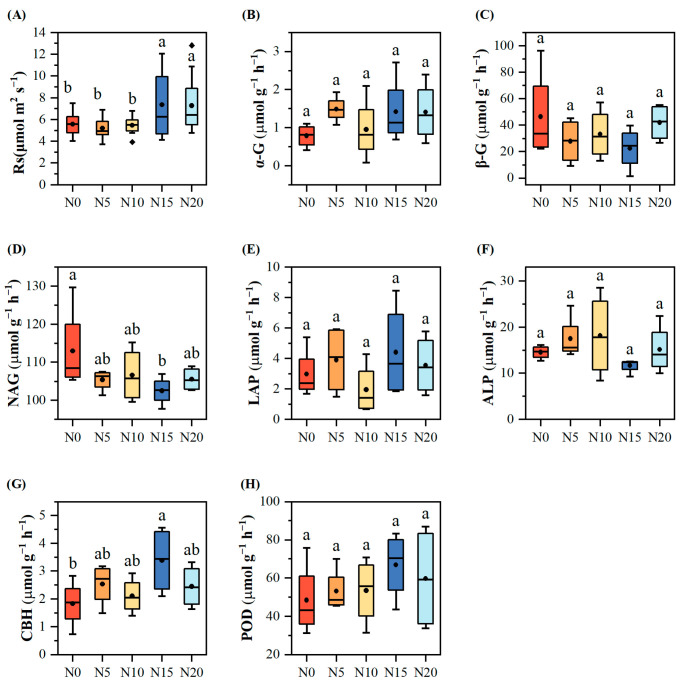
Analysis of soil respiration and soil enzyme activities at different levels of nitrogen addition. (**A**) Soil respiration (Rs); (**B**) α-1,4-glucosidase (α-G); (**C**) β-1,4-glucosidase (β-G); (**D**) N-acetylglucosaminidase (NAG); (**E**) L-leucine aminopeptidase (LAP); (**F**) Alkaline phosphatase (ALP); (**G**) Cellobiohydrolase (CBH); (**H**) Peroxidase (POD). Different letters indicate significant differences at *p* < 0.05.

Soil respiration rates at varying nitrogen addition levels were ordered as follows: N15 (7.36 ± 0.71 μmol m^2^ s^−1^) > N20 (7.21 ± 0.77 μmol m^2^ s^−1^) > N0 (5.58 ± 0.30 μmol m^2^ s^−1^) > N10 (5.47 ± 0.23 μmol m^2^ s^−1^) > N5 (5.20 ± 0.26 μmol m^2^ s^−1^). The highest soil respiration rate was observed for the N15 treatment (7.36 ± 0.71 μmol m^2^ s^−1^), which was 31.96% greater than that of the control (N0). Soil respiration rates differed significantly among nitrogen treatments (F_4,58_ = 3.92, *p* = 0.007). Post hoc comparisons indicated that high-nitrogen treatments (N15 and N20) increased soil respiration by approximately 33% relative to the other treatments (*p* < 0.05, Figure 2A). 

Regarding soil enzymes, no significant differences (*p* > 0.05) were observed in the activities of α-G, β-G, LAP, ALP, and POD among the treatments (Figure 2). However, the NAG content declined as the nitrogen input increased, reaching the highest value under the N0 treatment (113.01 ± 5.65 μmol g^−1^ h^−1^). In contrast, the CBH content generally increased, peaking at the N15 treatment (3.39 ± 0.61 μmol g^−1^ h^−1^), which was 85.07% higher than the control. The CBH levels in the N5, N10, and N20 treatments were 38.48%, 15.01%, and 33.71% higher than those of the control, respectively. Similarly, POD content exhibited a general upward trend.

**Figure 3:**
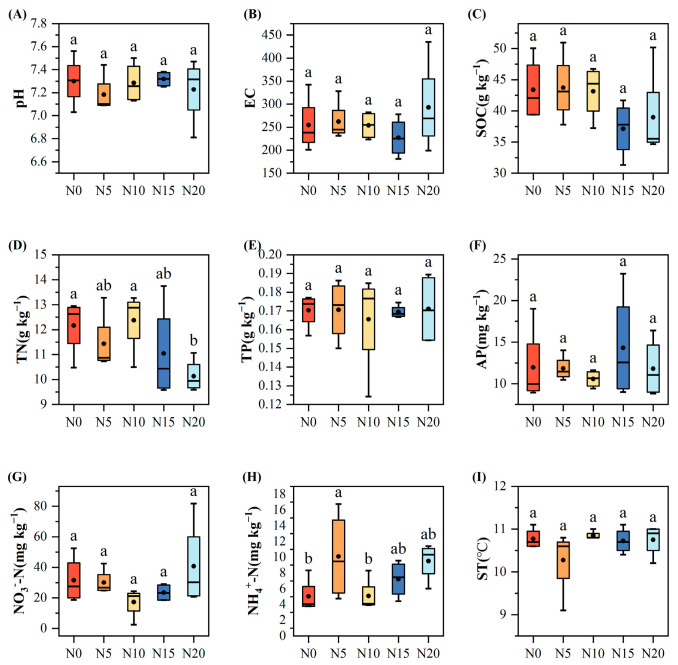
Analysis of soil physicochemical properties at different levels of nitrogen addition. (**A**) pH; (**B**) electrical conductivity (EC); (**C**) soil organic carbon (SOC); (**D**) total nitrogen (TN); (**E**) total phosphorus (TP); (**F**) available phosphorus (AP); (**G**) soil nitrate nitrogen (NO_3_^−^-N); (**H**) soil ammonium nitrogen (NH_4_^+^-N); (**I**) soil temperature (ST). Different letters indicate significant differences at *p* < 0.05.

The application of varying levels of nitrogen addition significantly affected certain soil physicochemical factors. ST, EC, pH, SOC, TP, AP, and NO_3_^−^-N did not differ significantly across the treatments (*p* < 0.05, Figure 3). With an increasing nitrogen addition, total nitrogen (TN) showed a dropping tendency. The N20 treatment recorded a TN content of 10.13 ± 0.33 g kg^−1^, which was 16.72% lower than that of N0 treatment and considerably lower than that of the N0 (12.17 ± 0.58 g kg^−1^, *p* = 0.045) and N10 treatments (12.38 ± 0.64 g kg^−1^, *p* = 0.029). Under the N5 treatment, the soil NH_4_^+^-N content reached its highest value of 10.10 ± 2.81 mg kg^−1^, which was 100.8% higher than the control and significantly higher than the N0 (5.03 ± 1.10 mg kg^−1^, *p* = 0.043) and N10 treatments (5.10 ± 1.06 mg kg^−1^, *p* = 0.045). Although SOC did not show statistically significant differences, it generally decreased as nitrogen addition increased.

### 3.2. Structure and Functional Features of Soil Microbial Communities Across Varying Nitrogen Addition Levels

**Figure 4:**
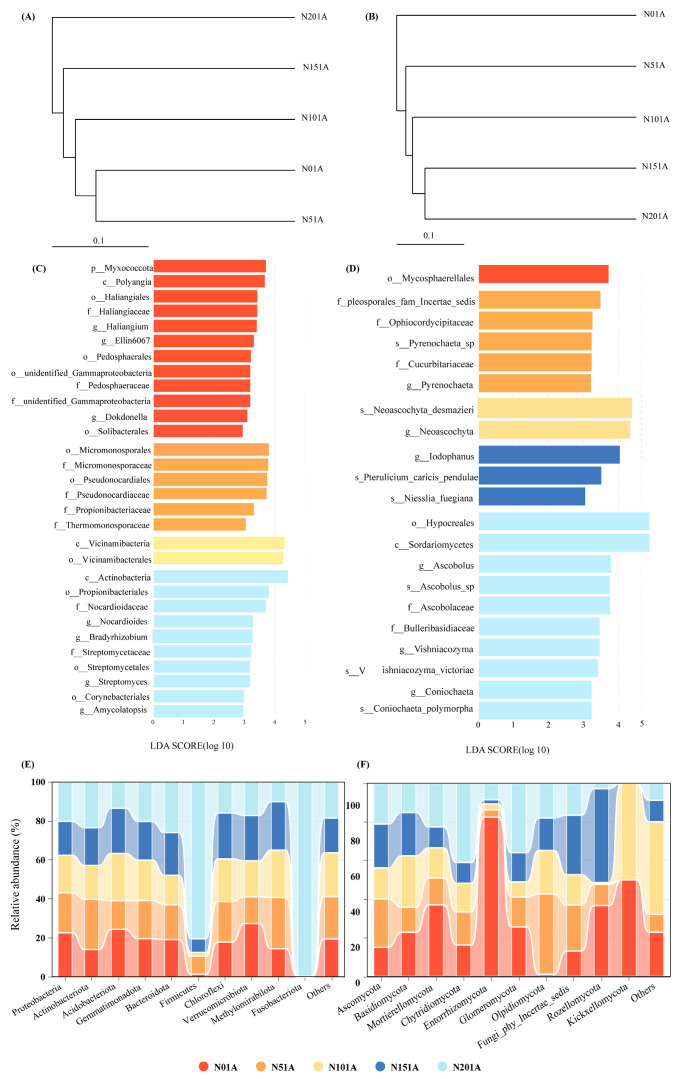
Effects of nitrogen addition on soil microbial communities. (**A**) UPGMA clustering of bacterial communities; (**B**) UPGMA clustering of fungal communities; (**C**) LEfSe analysis of bacterial communities (LDA > 3.5); (**D**) LEfSe analysis of fungal communities (LDA > 3.5); (**E**) Relative abundance of dominant bacterial phyla (Top 10); (**F**) Relative abundance of dominant fungal phyla (Top 10).

To explore the effects of nitrogen addition on soil microbial communities, UPGMA clustering based on Bray–Curtis distances was performed for bacterial and fungal communities (Figure 4A,B). The results showed that nitrogen addition altered microbial community structures, with both bacterial and fungal communities clustering into two distinct groups under high (N15, N20) and low (N0, N5) nitrogen treatments. LEfSe analysis further revealed significant differences in microbial taxa across treatments (Figure 4C,D). For bacteria, more indicator taxa were observed in N0 and N20 treatments. Proteobacteria-related genera such as Bradyrhizobium, together with Actinobacteria genera like Streptomyces and Amycolatopsis, were enriched under N20, while Myxococcota and Polyangia were dominant under N0. For fungi, the N20 treatment showed greater taxonomic differentiation, with enrichment of Hypocreales, Sordariomycetes, and *Ascobolus* (Ascomycota), whereas *Iodophanus* was specifically enriched under N15.

The top 10 phyla of the bacterial and fungal communities were chosen for investigation in this study (Figure 4 and Tables S2 and S3) based on the findings of the species annotation, while the remaining species were combined into the “others” category (Figure 4E,F). Nitrogen addition significantly affected microbial community composition at the phylum level. Bacterial communities were dominated across all nitrogen treatments by Proteobacteria (21–27%), Actinobacteriota (14–25%), Acidobacteriota (12–21%), and Gemmatimonadota (12–13%) (Figure 4E). Actinobacteriota abundance increased significantly under N addition (25% in N5, 23% in N20), while Acidobacteriota showed the opposite trend, with the highest abundance under N0 (21%) and the lowest under N20 (11%). Fungal communities, regardless of nitrogen treatment level, were consistently predominated by Ascomycota (44–73%), Basidiomycota (13–26%), and Mortierellomycota (6–21%) (Figure 4F). Chytridiomycota reached its highest abundance (6%) under N20, two to three times higher than in other treatments.

**Figure 5:**
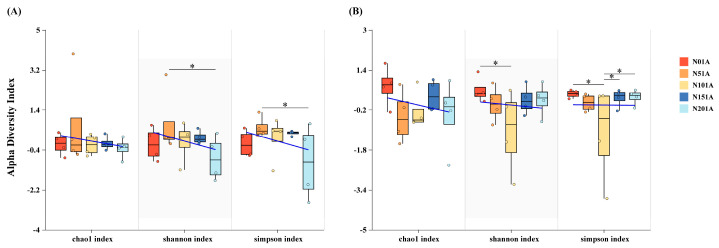
Effect of different levels of nitrogen addition on soil microbial alpha diversity. (**A**) bacterial community alpha diversity index; (**B**) fungal community alpha diversity index, * *p* < 0.05.

The addition of nitrogen had a significant impact (*p* < 0.05) on the richness indices (Shannon and Simpson) of both bacterial and fungal communities. There were no significant differences in the chao1 index of bacteria and fungi between treatments. The Shannon and Simpson indices exhibited a notable decline under the high nitrogen addition (N20) treatment relative to the N5 treatment (*p* < 0.05). The fungal community had the lowest Shannon and Simpson indices under the N10 treatment, indicating a decrease in diversity, which increased as nitrogen levels rose. Compared to other treatments, fungal richness in the N10 group was significantly reduced (*p* < 0.05, Figure 5).

**Figure 6:**
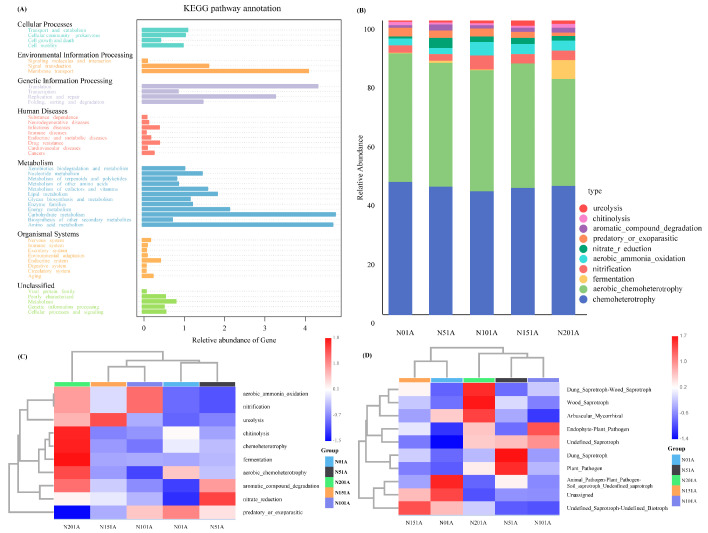
Predicted functional profiles of soil microbial communities under different nitrogen addition levels. (**A**) Functional prediction of bacterial communities based on Tax4Fun (Level 2 KEGG pathways); (**B**) Top 10 predicted bacterial functions based on FAPROTAX; (**C**) Heatmap of predicted bacterial functions across treatments based on FAPROTAX; (**D**) Heatmap of predicted fungal ecological guilds across treatments based on FUNGuild.

Functional profiles of soil bacteria and fungi were predicted using Tax4Fun and FAPROTAX. Based on KEGG annotations (Figure 6A–C; Table S4), bacterial functions were categorized into seven major Level 1 KEGG functional groups. The dominant functions were Metabolism (47.7–49.0%), followed by Genetic Information Processing (21.1–23.3%) and Environmental Information Processing (11.9–13.5%). Among Level 2 functions, Carbohydrate metabolism and Amino acid metabolism were most abundant (22.3–24.5%), along with Membrane transport (19.1–21.4%) and Translation (19.2–24.9%) as key components. Bacterial metabolic functions varied significantly across nitrogen addition treatments. The N0 treatment exhibited generally lower metabolic activity, while N20 showed the most active metabolic profile, with marked enrichment in chemoheterotrophy, aerobic chemoheterotrophy, and fermentation. Notably, fermentation reached its highest level under N20 (2.7%). The function ureolysis peaked under the N15 treatment. Fungal functional guilds, predicted by FUNGuild (Figure 6D; Table S5), were dominated by Unassigned taxa (with the highest relative abundance in N0 at 63.73%) and Undefined Saprotrophs (peaking in N10 at 41.83%). In contrast, Wood Saprotrophs, Dung Saprotroph–Wood Saprotrophs, and Arbuscular Mycorrhizal fungi were enriched under the N20 treatment.

### 3.3. Soil Respiration and Its Relationships with Soil Environmental Factors, Enzyme Activities, and Soil Microbial Communities

**Figure 7:**
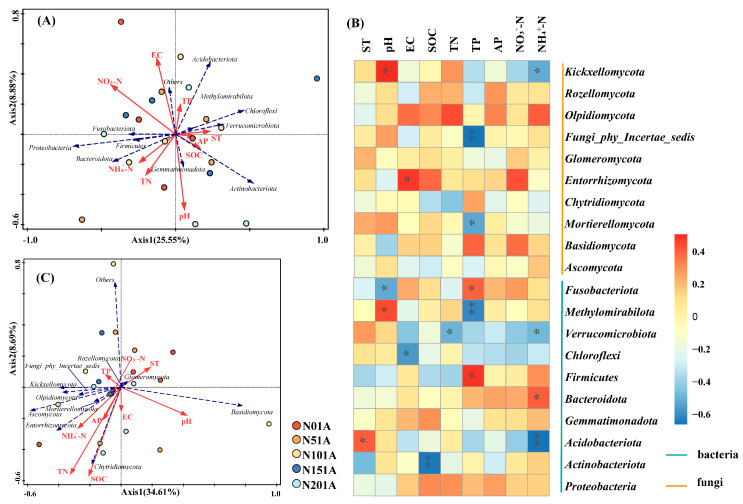
Relationships between soil microbial communities and environmental factors under different nitrogen addition levels. (**A**) Redundancy analysis (RDA) of bacterial communities and soil environmental factors; (**B**) Heatmap of Spearman correlations between dominant microbial phyla (Top 10) and soil physicochemical properties; (**C**) Redundancy analysis (RDA) of fungal communities and soil environmental factors. ** *p* < 0.01, * *p* < 0.05. Detailed correlation coefficients are provided in Supplementary Table S6.

The effects of soil environmental factors on microbial communities varied depending on the microbial taxon. The relationships between the top 10 bacteria and fungi in terms of phylum-level abundance and soil environmental factors are shown in Figure 7. The explanation rates of the first and second axes at the phylum level were 25.55% and 8.88%, respectively. The first two axes explained a total of 34.43% of the variance in the bacterial community (Figure 7A). The outcomes demonstrated that the main environmental variables influencing the bacterial community were NO_3_^−^-N, TN, and SOC. Positive relationships were observed between TN and NH_4_^+^-N, while NO_3_^−^-N showed a negative correlation with SOC. Proteobacteria and Bacteroidota were positively correlated with TN, NH_4_^+^-N and NO_3_^−^-N; while Actinobacteria were positively related to SOC. As shown in Figure 7B, the first two axes accounted for 43.3% of the variance in the fungal community, while the first and second axes explained 34.61% and 8.69%, respectively. NH_4_^+^-N, and TN were the principal environmental factors influencing the fungal community. NH_4_^+^-N is a key factor influencing fungal community structure (13.2% variance). Chytridiomycota exhibited a positive correlation with SOC, while Ascomycota and Entorrhizomycota showed positive correlations with both NH_4_^+^-N and TN.

Spearman correlation analysis was performed between the top 10 most abundant microbial phyla and soil physicochemical properties (Figure 7B). The results revealed distinct responses of bacterial and fungal taxa to environmental factors. Actinobacteriota showed a significant negative correlation with SOC (*p* < 0.01), while Acidobacteriota was negatively correlated with NH_4_^+^-N (*p* < 0.01), and Bacteroidota was positively correlated with NH_4_^+^-N (*p* < 0.05). Mortierellomycota exhibited a significant negative correlation with TP (*p* < 0.05), and Fungi_phy_Incertae_sedis was also suppressed by TP (*p* < 0.01). Methylomirabilota was negatively correlated with TP (*p* < 0.01) but positively correlated with soil pH (*p* < 0.05). Complete Spearman correlation matrices for microbial phyla are available in Supplementary Table S6.

**Figure 8:**
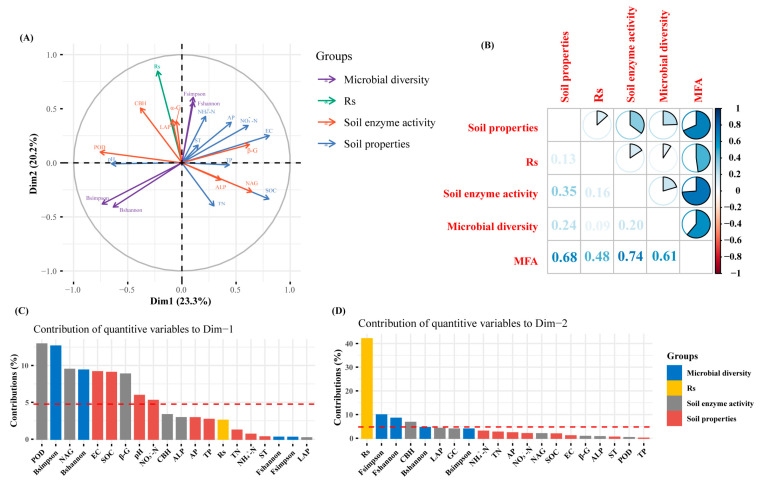
Multiple relationship tests of soil enzyme activities, soil physicochemical properties, and soil microbial diversity with soil respiration (**A**) MFA factorial map of variable groups projected onto the first two dimensions. (purple line: microbial diversity; Fshannon: soil fungal Shannon index; Fsimpson: fungal Simpson index; green line: soil respiration (Rs); red line: soil enzyme activity, includingα-G, β-G, CBH, NAG, LAP, ALP, and POD; blue line: soil physicochemical properties, including ST, pH, EC, TN, TP, AP, SOC, NO_3_^−^-N, and NH_4_^+^-N. (**B**) RV coefficient–based correlation plot of variable groups. (**C**) Percentage contributions of each quantitative variable to Dimension 1. (**D**) Percentage contributions of each quantitative variable to Dimension 2.

The MFA revealed that soil respiration was influenced by various environmental factors, enzyme activities, and microbial communities (Figure 8). The first and second axes explained 43.5% of the total variance in soil respiration, with contributions of 23.3% and 20.2%, respectively. The RV coefficients of the MFA for soil respiration, soil enzyme activity, soil physicochemical factors, and soil microbial diversity were 0.48, 0.74, 0.68, and 0.61, respectively. Soil respiration showed positive correlations with NH_4_^+^-N, ST, NO_3_^−^-N, pH, α-G, LAP, CBH, and POD, and negative correlations with SOC, TN, and NAG. Moreover, it was positively associated with fungal diversity but negatively correlated with bacterial diversity. Fungal diversity and soil physicochemical parameters (apart from TN, SOC, pH, and TP) showed a positive correlation.

Partial least squares path modeling (PLS-PM) demonstrated that nitrogen addition explained 78% of the variance in soil respiration (R^2^ = 0.78, Figure S1). Nitrogen addition exerted a significant positive direct effect on soil respiration (β = 0.75, *p* = 0.002) and also had indirect effects through alterations in the microbial community and soil physicochemical properties. Nitrogen addition reduced bacterial diversity (β = –0.64, *p* = 0.004) and fungal diversity (β = –0.14, *p* = 0.624). Soil physicochemical properties significantly affected soil enzyme activities (β = –0.69, *p* < 0.001). Overall, nitrogen addition altered soil respiration primarily through direct effects and microbially mediated pathways.

## 4. Discussion

### 4.1. Nitrogen Addition Modulates Rs by Functionally Restructuring Soil Microbial Guilds

High nitrogen addition significantly promoted Rs, with the highest value (7.36 μmol m^−2^ s^−1^) recorded under the N15 treatment—a 31.96% increase over the N0 control. This Rs enhancement was associated with substantial shifts in microbial community structure and function. UPGMA clustering and LEfSe analysis revealed that nitrogen addition dramatically restructured microbial communities, with high-N treatments (N15/N20) enriching *Proteobacteria* (e.g., *Bradyrhizobium*) and 12 prototrophic fungal taxa. *Actinobacteriota* emerged as a key cellulose-degrading phylum [40], while *Ascomycota* (44–73%) and *Basidiomycota* (13–26%) were consistently predominant fungal phyla across all nitrogen treatments, exhibiting robust organic matter decomposition and nitrogen metabolism. These phyla facilitate the transformation of litter carbon into microbial residues and the processing of recalcitrant substrates such as cellulose and lignin [41,42]. Concomitantly, elevated POD activity may have contributed to enhanced CO_2_ release. Notably, the coprophilous saprotroph *Iodophanus* was enriched in N15 plots, a genus reported in previous studies to thrive in dung-rich environments and exhibit cellulolytic and lignolytic capacities [43]. Its enrichment was associated with enhanced degradation potential for plant-derived carbon [44], aligning precisely with the observed peak in CBH activity and maximum Rs under the N15 treatment.

Furthermore, functional prediction analyses elucidated the impact of nitrogen addition on microbial community functions, revealing that core functions centered on energy and nutrient metabolism. Tax4Fun and FAPROTAX analyses indicated that carbon metabolism functions—Carbohydrate metabolism, Amino acid metabolism, and fermentation—were dominant in the bacterial community. These two carbon-cycling-related functions are well-known to be associated with the degradation of complex compounds and energy flow for microbial growth and development, respectively. Their enrichment suggests that nitrogen addition may potentially influence soil carbon storage and CO_2_ fluxes by altering the soil microbial community [18,45]. Ureolysis activity peaked under the N15 treatment, suggesting microbes in this treatment likely enhanced nitrification processes via urea hydrolysis, thereby increasing soil NH_4_^+^-N content [46]. Shifts in fungal ecological guilds, such as saprotrophic fungi accelerating litter decomposition and mycorrhizal fungi contributing to root-derived carbon input, have been reported to significantly influence soil organic carbon dynamics [18,44]. FunGuild predictions demonstrated that fungal functional groups such as Wood Saprotrophs and Arbuscular Mycorrhizal fungi became markedly dominant under high-N treatments. This shift, concomitant with rising cellulase activity and increased NH_4_^+^-N content, accelerated organic matter decomposition and CO_2_ release, thereby propelling the observed increase in soil respiration [44,47]. However, it should be noted that these inferences rely on marker gene-based prediction algorithms rather than direct metagenomic or metatranscriptomic measurements. As such, uncertainties due to reference genome biases must be considered, and the functional assignments should be interpreted with caution. Therefore, our discussion of functional shifts should be viewed as suggesting putative metabolic potentials that highlight hypotheses for future validation through direct ‘omics’ approaches.

### 4.2. Multifactor Regulation of the Rs Response to Nitrogen Addition

Soil respiration (Rs) is a complex process jointly regulated by multiple biotic and abiotic factors. In addition to microbial community shifts, temperature fluctuations, soil moisture dynamics, root respiration all play important roles in controlling soil CO_2_ efflux [10,13]. Previous studies have shown that these factors interact in non-linear ways, and thus Rs cannot be fully explained by single drivers or simple linear relationships [37]. In our study, although nitrogen addition significantly influenced microbial community composition and enzyme activities, these biological changes likely operated in concert with abiotic constraints such as seasonal variations in temperature and moisture availability [6].

A meta-analysis of alpine meadows on the Tibetan Plateau confirms that nitrogen addition elevates soil NH_4_^+^-N content [48]. Crucially, the effect of nitrogen addition on Rs is nitrogen-form-dependent [13]. In this study, where NH_4_NO_3_ served as the sole nitrogen source, it stimulated soil microbial metabolism and accelerated the mineralization of soil organic matter, releasing readily bioavailable NH_4_^+^-N [49].

Enzymatic reactions bridge soil physicochemical properties and microbial metabolic shifts. Multiple Factor Analysis (MFA) identified strong positive correlations between Rs and enzyme activities, including CBH, LAP, and POD, confirming that soil respiration fundamentally relies on efficient microbial decomposition of organic substrates mediated by enzymatic catalysis. NAG is a nitrogen-degrading hydrolase, and CBH is a carbon-degrading hydrolase [50]. In accordance with the tenets of resource allocation theory, increasing soil effective nitrogen can meet the N demand of microorganisms and decrease the activity of nitrogen-degrading hydrolases, which in turn increases the activity of carbon-degrading hydrolases to balance the availability of soil nutrients [51]. The addition of nitrogen has been demonstrated to exert a direct or indirect influence on the decomposition and mineralization of SOC through the actions of CBH and POD enzymes [22,52]. Furthermore, the activity of POD has been shown to impact the redox reactions occurring within the soil, which in turn stimulates soil respiration [11]. Their interactions with nitrogen availability explain the significant increase in respiration observed under nitrogen treatments.

The moderate explanatory power of RDA (34–43%) reflects inherent challenges in partitioning microbial community variation, where unmeasured biotic interactions (e.g., microbial competition/predation) and micro-environmental heterogeneity (e.g., soil micropore dynamics) likely contribute significantly [39]. PLS-PM modeling quantified that nitrogen addition significantly enhances Rs (R^2^ = 0.78) by regulating physicochemical properties, restructuring microbial communities and functions, and elevating key enzyme activities. Critically, fungi exerted a significantly stronger positive effect on Rs than bacteria, likely attributable to the dominant role of fungal-driven saprotrophic pathways in carbon cycling [29]. Although most studies emphasize abiotic drivers of Rs [10], our study demonstrates that microbial community structure and function are key mediators of ecosystem carbon fluxes under nitrogen deposition.

Natural nitrogen deposition is a long-term process; key non-growing season processes—such as winter soil respiration under snow cover [53], early spring freeze–thaw pulses [54], and late autumn microbial activity [55]—were not included. Therefore, the cross-seasonal stability and long-term persistence of microbial community restructuring and functional succession remain to be validated through future long-term investigations. In future investigations, it is advisable to administer nitrogen in multiple small doses to simulate natural deposition, coupled with continuous monitoring over an annual or multi-seasonal timeframe, thereby enabling a more comprehensive quantification of soil respiration dynamics and the interactions between nitrogen enrichment and other environmental variables at the scale of a year.

## 5. Conclusions

In this study, we examined the factors influencing soil respiration under varying levels of N addition, using NH_4_NO_3_ as the sole nitrogen source. The results revealed a nonlinear response of Rs to N input, with high N addition significantly enhancing soil respiration. Alterations in microbial community structure and function under N enrichment were associated with Rs variation, primarily by increasing soil available nitrogen, which in turn accelerated the mineralization of soil organic matter and provided substrates for microbial activity and soil enzyme function. Partial least squares path modeling (PLS-PM) indicated that nitrogen addition influenced Rs both directly and indirectly, primarily via alterations in soil physicochemical characteristics, enhanced enzyme activities, and changes in microbial community structure and functional traits. Notably, fungi exhibited a stronger positive association with Rs. These findings provide valuable short-term insights into carbon balance and the sustainable management of grassland ecosystems in this region while highlighting the need for long-term studies to confirm the persistence of these relationships.

## Data Availability

The original data presented in the study are openly available in National Center for Biotechnology Information at PRJNA1176033.

## Supplementary Materials

The following supporting information can be downloaded at: https://www.mdpi.com/article/10.3390/microorganisms13092098/s1, Table S1. Soil enzyme functions and corresponding substrates. Table S2. Relative abundance of the top 10 species at the bacterial community phylum level. Table S3. Relative abundance of the top 10 species at the fungal community phylum level. Table S4. Top 10 predicted bacterial functional groups based on FAPROTAX. Table S5. Top 10 predicted fungal functional guilds based on FUNGuild. Table S6. Spearman correlations between soil microbial phyla and environmental factors. Significant correlations are highlighted in bold, with asterisks indicating levels of statistical significance (* *p* < 0.05, ** *p* < 0.01, *** *p* < 0.001). Figure S1. Direct and indirect effects of N addition, soil physicochemical properties, soil enzyme activities, and soil microbes on soil respiration based on partial least squares path modeling (PLS-PM). Numbers next to the paths are standardized path coefficients, path widths are scaled according to the size of the standardized path coefficients, solid and dashed lines represent positive and negative paths, respectively, *** *p* < 0.001, ** *p* < 0.01, * *p* < 0.05. Goodness-of-fit of the model (GOF) = 0.53.

## Abbreviations

The following abbreviations are used in this manuscript:

α-G	α-1,4-glucosidase
β-G	β-1,4-glucosidase
ALP	Alkaline phosphatase
AP	Available phosphorus
CBH	Cellobiohydrolase
EC	Electrical conductivity
LAP	L-leucine aminopeptidase
LSD	Least significant difference
MFA	Multiple factor analysis
NAG	N-acetylglucosaminidase
NH_4_^+^-N	Soil ammonium nitrogen
NO_3_^−^-N	Soil nitrate nitrogen
PLS-PM	Partial least squares path modeling
POD	Peroxidase
Rs	Soil respiration
RDA	Redundancy analysis
TN	Total nitrogen
SOC	Soil organic carbon
ST	Soil temperature
