# Supplementary material for: Microbial Communities and Environmental Factors Interact to Regulate Soil Respiration Under Nitrogen Addition Conditions in Alpine Meadows in Northwest China
Source: Microorganisms. 2025 Sep 9;13(9):2098. doi: 10.3390/microorganisms13092098 (PMC12472389; doi:10.3390/microorganisms13092098)
Supplement: Supplementary file 1 [file microorganisms-13-02098-s001.zip › microorganisms-3797407-supplementary.pdf]

Microbial communities and environmental factors interact to regulate soil respiration under nitrogen addition conditions in alpine meadows in northwest China

Xiaojuan Cao[1,2,3], Jinlong Wang[1,2,3*], Bota Bahethan[1,2,3], Yudong Chen[1,2,3], Junjie Liu[1,2,3] and Guanghui Lü[1,2,3]

[1]College of Ecology and Environment, Xinjiang University, Urumqi, 830017, China

[2]Key Laboratory of Oasis Ecology, Ministry of Education (Xinjiang University), Urumqi, 830017, China

[3]Xinjiang Jinghe Observation and Research Station of Temperate Desert Ecosystem, Ministry of Education, Börtala Mongol Autonomous Prefecture, 833300, China

Correspondence to: Jinlong Wang (wangjl@xju.edu.cn)

Supplementary Material

Table S1. Soil enzyme functions and corresponding substrates.

Enzyme	Abbreviation	Function	Substrate
α-1, 4-glucosidase	α-G	Carbon cycle. Hydrolysis of soluble sugars to glucose.	4-MUB-α-D-glucoside
β-1, 4-glucosidase	β-G	Carbon cycle. Releases glucose from cellulose.	4-MUB-β-D-glucoside
Cellobiohydrolase	CBH	Carbon cycle. Hydrolysis of cellulose to disaccharides.	4-MUB-β-D-cellobioside
N-acetylglucosaminidase	NAG	Nitrogen cycle. Hydrolysis of chitin.	4-MUB-N-acetyl-β-D-glucosaminide
L-leucine aminopeptidase	LAP	Nitrogen cycle. Hydrolysis of leucine and other hydrophobic amino acids.	L-Leucine-7-amino-4-methylcoumarin
Alkaline phosphatase	ALP	Phosphorus cycle. Hydrolysis of phosphoglycans and phospholipids.	4-Methylumbelliferone phosphate
peroxidase	POD	Degradation of lignin and catalytic oxidation reactions.	Levodopa and sodium EDTA

Table S2. Relative abundance of the top 10 species at the bacterial community phylum level.

Taxonomy	Different treatments				
	**N01A**	**N51A**	**N101A**	**N151A**	**N201A**
Proteobacteria	0.270706	0.244279	0.230061	0.208587	0.24227
Actinobacteriota	0.138472	0.253772	0.1706	0.189452	0.231595
Acidobacteriota	0.212561	0.127664	0.211453	0.201209	0.118022
Gemmatimonadota	0.123226	0.122629	0.130647	0.123913	0.12752
Bacteroidota	0.054107	0.049664	0.042697	0.061634	0.073183
Firmicutes	0.000607	0.003692	0.000847	0.002941	0.033166
Chloroflexi	0.068533	0.079991	0.084354	0.090033	0.061554
Verrucomicrobiota	0.05497	0.027429	0.037108	0.04633	0.03453
Methylomirabilota	0.008023	0.014559	0.013424	0.013744	0.005599
Fusobacteriota	0	0.000021	0	0	0.007714
Others	0.068794	0.0763	0.078809	0.062156	0.064847

Table S3. Relative abundance of the top 10 species at the fungal community phylum level.

Taxonomy	Different treatments				
	**N01A**	**N51A**	**N101A**	**N151A**	**N201A**
Ascomycota	0.439536	0.728835	0.466038	0.658364	0.614681
Basidiomycota	0.224258	0.127519	0.260384	0.219346	0.148894
Mortierellomycota	0.21316	0.079702	0.08956	0.062092	0.129931
Chytridiomycota	0.024692	0.026045	0.022616	0.01637	0.062479
Entorrhizomycota	0.013435	0.000616	0.000522	0.000378	0.001395
Glomeromycota	0.009069	0.005407	0.002822	0.005258	0.012577
Fungi_phy_Incertae_sedis	0.000457	0.000816	0.000536	0.001166	0.000592
Olpidiomycota	0.000028	0.000592	0.000317	0.000247	0.000257
Rozellomycota	0.000201	0.000079	0.000019	0.000266	0.000014
Kickxellomycota	0.000047	0	0.000023	0	0
Others	0.075117	0.030388	0.157161	0.036513	0.02918

Table S4. Top 10 predicted bacterial functional groups based on FAPROTAX.

feature	Different treatments				
	**N201A**	**N01A**	**N51A**	**N151A**	**N101A**
chemoheterotrophy	0.183123	0.148857	0.142023	0.137355	0.132504
aerobic_chemoheterotrophy	0.15204	0.144355	0.136542	0.134292	0.129159
fermentation	0.027255	0.000627	0.001985	0.000514	0.000761
nitrification	0.013693	0.007688	0.007054	0.010373	0.014958
aerobic_ammonia_oxidation	0.013693	0.007688	0.007054	0.010373	0.014958
nitrate_reduction	0.007397	0.002671	0.0113	0.006582	0.00545
predatory_or_exoparasitic	0.004452	0.009599	0.008347	0.006622	0.008616
aromatic_compound_degradation	0.006567	0.002938	0.006077	0.004363	0.003663
chitinolysis	0.005857	0.0036	0.002603	0.00234	0.002481
ureolysis	0.003701	0.000947	0.001345	0.00501	0.001823
Others	0.582222	0.671031	0.67567	0.682177	0.685628

Table S5. Top 10 predicted fungal functional guilds based on FUNGuild.

feature	Different treatments				
	**N51A**	**N151A**	**N01A**	**N201A**	**N101A**
Unassigned	0.48114	0.57621	0.63727	0.44796	0.47943
Undefined_Saprotroph	0.39667	0.30559	0.25808	0.39131	0.41831
Endophyte-Plant_Pathogen	0.01221	0.02631	0.00582	0.03663	0.05038
Plant_Pathogen	0.03645	0.00590	0.00282	0.01898	0.01077
Animal_Pathogen-Plant_Pathogen-Soil_Saprotroph-Undefined_Saprotroph	0.02062	0.01356	0.03443	0.00958	0.01178
Undefined_Saprotroph-Undefined_Biotroph	0.00087	0.03186	0.02222	0.01104	0.00023
Wood_Saprotroph	0.00453	0.00380	0.00042	0.01532	0.00100
Arbuscular_Mycorrhizal	0.00541	0.00530	0.00899	0.01264	0.00284
Dung_Saprotroph-Wood_Saprotroph	0.00028	0.00510	0.0000046	0.01145	0.00310
Dung_Saprotroph	0.00761	0.00232	0.00055	0.00202	0.00145
Others	0.03417	0.02400	0.02935	0.04301	0.02065

Table S6. Spearman correlations between soil microbial phyla and environmental factors. Significant correlations are highlighted in bold, with asterisks indicating levels of statistical significance (**P* < 0.05, ***P* < 0.01, ****P* < 0.001).

Taxonomy	Factor								
	ST	pH	EC	SOC	TN	TP	AP	NO_3^--N	NH_4^+-N
**Bacteria**									
**Proteobacteria**	-0.14	-0.17	0.22	0.37	0.35	0.25	0.32	0.2	0.21
**Actinobacteriota**	-0.43	0.14	-0.07	**-0.61****	-0.16	0.05	-0.25	0.11	0.2
**Acidobacteriota**	0.46	0.13	-0.28	-0.13	0.09	-0.33	-0.17	-0.38	**-0.63****
**Gemmatimonadota**	-0.06	0.13	0.26	0.36	-0.02	-0.16	0.15	0.17	0.25
**Bacteroidota**	-0.12	-0.09	0.13	-0.03	0.03	0.26	0.31	0.3	**0.46***
**Firmicutes**	-0.34	-0.26	0.11	-0.32	-0.35	**0.54***	0.05	0.1	0.36
**Chloroflexi**	0.14	0.1	**-0.52***	-0.16	0.04	-0.29	-0.31	-0.35	-0.26
**Verrucomicrobiota**	0.33	0.18	-0.4	-0.13	**-0.46***	-0.34	-0.27	-0.39	**-0.45***
**Methylomirabilota**	-0.03	**0.50***	-0.17	0.07	0.19	**-0.58****	0.07	-0.02	0.15
**Fusobacteriota**	-0.22	**-0.48***	0.3	0.16	-0.21	**0.46***	0.27	0.33	0.16
**Fungi**									
**Ascomycota**	-0.16	0.09	-0.24	-0.3	-0.1	-0.26	-0.04	-0.11	0.17
**Basidiomycota**	0.01	-0.39	0.15	0.15	-0.12	0.39	0.09	0.37	0.14
**Mortierellomycota**	0.24	0.28	-0.08	0.12	-0.04	-0.53*	0.04	-0.13	-0.18
**Chytridiomycota**	-0.16	-0.05	-0.06	-0.35	-0.44	0.25	-0.23	-0.08	-0.25
**Entorrhizomycota**	0.1	-0.25	**0.47***	0.39	0.07	0.11	0.14	0.42	-0.1
**Glomeromycota**	0.22	-0.09	0.03	0.11	0.02	-0.17	-0.21	0.16	0
**Fungi_phy_Incertae_sedis**	0.05	0.27	-0.28	0.05	-0.09	**-0.66*****	-0.01	-0.04	0.1
**Olpidiomycota**	-0.24	0.11	0.37	0.31	0.44	-0.02	0.32	0.08	0.41
**Rozellomycota**	-0.17	0.12	-0.29	0.23	0.22	-0.31	0.29	0.06	0.09
**Kickxellomycota**	0.11	**0.50***	-0.17	-0.04	0.29	-0.35	-0.15	-0.38	**-0.52***

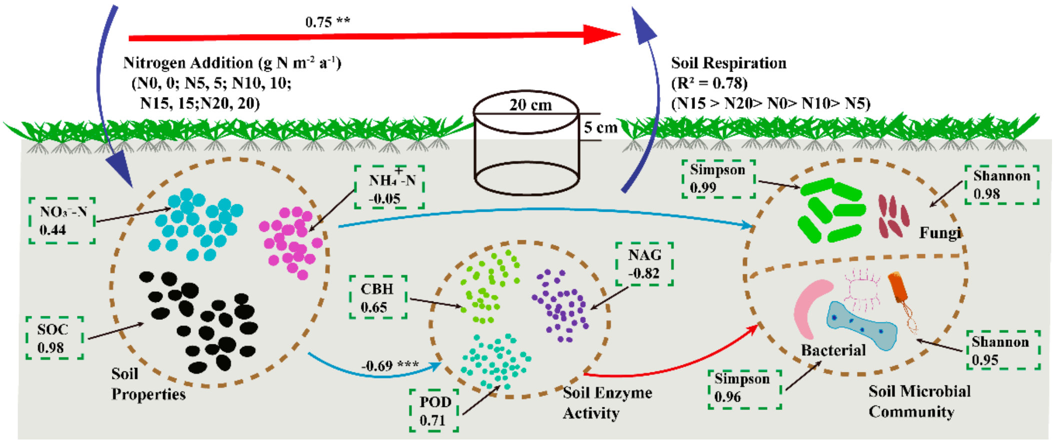

Figure S1. Direct and indirect effects of N addition, soil physicochemical properties, soil enzyme activities, and soil microbes on soil respiration based on partial least squares path modeling (PLS-PM). Numbers next to the paths are standardized path coefficients, path widths are scaled according to the size of the standardized path coefficients, solid and dashed lines represent positive and negative paths, respectively, ***P < 0.001, **P < 0.01, *P < 0.05. Goodness-of-fit of the model (GOF) = 0.53.
